# Ceramide Synthase 6 Is a Novel Target of Methotrexate Mediating Its Antiproliferative Effect in a p53-Dependent Manner

**DOI:** 10.1371/journal.pone.0146618

**Published:** 2016-01-19

**Authors:** Baharan Fekry, Amin Esmaeilniakooshkghazi, Sergey A. Krupenko, Natalia I. Krupenko

**Affiliations:** 1 Nutrition Research Institute, UNC Chapel Hill, Kannapolis, NC, United States of America; 2 Department of Nutrition, UNC Chapel Hill, Chapel Hill, NC, United States of America; Université PARIS- DIDEROT (7), FRANCE

## Abstract

We previously reported that ceramide synthase 6 (CerS6) is elevated in response to folate stress in cancer cells, leading to enhanced production of C_16_-ceramide and apoptosis. Antifolate methotrexate (MTX), a drug commonly used in chemotherapy of several types of cancer, is a strong inhibitor of folate metabolism. Here we investigated whether this drug targets CerS6. We observed that CerS6 protein was markedly elevated in several cancer cell lines treated with MTX. In agreement with the enzyme elevation, its product C_16_-ceramide was also strongly elevated, so as several other ceramide species. The increase in C_16_-ceramide, however, was eliminated in MTX-treated cells lacking CerS6 through siRNA silencing, while the increase in other ceramides sustained. Furthermore, the siRNA silencing of CerS6 robustly protected A549 lung adenocarcinoma cells from MTX toxicity, while the silencing of another ceramide synthase, CerS4, which was also responsive to folate stress in our previous study, did not interfere with the MTX effect. The rescue effect of CerS6 silencing upon MTX treatment was further confirmed in HCT116 and HepG2 cell lines. Interestingly, CerS6 itself, but not CerS4, induced strong antiproliferative effect in several cancer cell lines if elevated by transient transfection. The effect of MTX on CerS6 elevation was likely p53 dependent, which is in agreement with the hypothesis that the protein is a transcriptional target of p53. In line with this notion, lometrexol, the antifolate inducing cytotoxicity through the p53-independent mechanism, did not affect CerS6 levels. We have also found that MTX induces the formation of ER aggregates, enriched with CerS6 protein. We further demonstrated that such aggregation requires CerS6 and suggests that it is an indication of ER stress. Overall, our study identified CerS6 and ceramide pathways as a novel MTX target.

## Introduction

Antifolates, a class of drugs mimicking the structure of folate coenzymes and therefore inhibiting folate enzymes, have been used for the treatment of malignancies for decades [1–3]. Methotrexate (MTX, amethopterin), a prototypical member of this group of drugs, was introduced for treatment of cancer in late 1940s [4]. Though since that time numerous novel antifolates with different mechanisms of action and activities have been developed, MTX is still commonly used as a chemotherapeutic [5, 6] and also for the treatment of rheumatoid arthritis [7]. The primary target of MTX is dihydrofolate reductase (DHFR), an enzyme converting dihydrofolate produced in the biosynthesis of thymidylate, back to the active form of folate, tetrahydrofolate [6, 8]. The enzyme also incorporates dietary folic acid into the reduced (active) intracellular folate pool [9]. Another direct target of MTX is thymidylate synthase (TYMS), the enzyme responsible for the thymidylate biosynthesis [3, 10]. Though MTX itself is a week inhibitor of TYMS, its polyglutamylated forms, generated in the cell, have much stronger effect on the enzyme with K_i_ of about 50 nM [11]. The inhibition of DHFR and TYMS depletes intracellular nucleotide pools, that is a general stress stimulus, and therefore the downstream effects are pleiotropic and involve diverse signaling pathways. Thus targets, which could be activated as the cellular response to MTX, include p53, HDAC, JNK, Bcl-2 family members, FAS, and caspases [12–17]. Of note, antifolates can employ additional mechanisms of cytotoxicity such as enhancement of autophagy and autophagy-dependent tumor cell killing [18].

Another cellular pathway implicated in antifolate action is ceramide signaling. Ceramides, a group of sphingolipids, are common structural components of cellular membranes [19]. Importantly, in recent years the function of ceramides as signaling molecules has been widely established [20–22]. As such, ceramides are involved in the regulation of major cellular processes including proliferation, differentiation, apoptosis, autophagy, senescence, and general response to various stress stimuli [20–23]. The first report connecting antifolates to ceramide pathways has demonstrated that the inhibitor of TYMS GW1843 activated ceramide-generating enzymes and lead to ceramide elevation in Molt-4 human T-cell leukemia cells [24]. Not much progress has been made since that time towards understanding the role of antifolates in the ceramide regulation. One report though indicated that the treatment of BT474 human mammary gland cells with Pemetrexed, an antifolate targeting multiple folate enzymes, in combination with sorafenib, a kinase inhibitor, resulted in increased C_16:0_; C_24:0_ and C_24:1_ dihydroceramide levels [25]. This study also underscored the role of ceramide synthase 6 (CerS6) in the generation of C_16:0_ dihydroceramide in response to Pemetrexed and sorafenib.

We have previously reported that folate stress induced by the expression of the folate enzyme ALDH1L1 or by folate starvation leads to ceramide elevation in A549 and HCT116 cell lines [26]. Furthermore, the induction of cytotoxicity by ALDH1L1 in these cells, as well as C_16_-ceramide accumulation, was CerS6 dependent. Overall, recently ceramide synthases have attracted attention as potential targets for therapeutic intervention [27]. In the present study, we have investigated CerS6 as a target of MTX.

## Materials and Methods

### Cell culture and reagents

Cell culture media and reagents were purchased from Invitrogen (Waltham, MA) and Corning (Cellgro, Manassas, VA). Cell lines were obtained from American Type Culture Collection. Generation of A549 cells with p53 silenced by shRNA was described previously [28]. HCT116 and HCT116 *p53*^−/−^ cell lines were a kind gift from Dr. Bert Vogelstein (The Sidney Kimmel Comprehensive Cancer Center, John Hopkins University School Medicine). Methotrexate (MTX) was purchased from Sigma Aldrich (St. Louis, MO). **Natural C**_**16**_-**ceramide was from Avanti Polar Lipids (Alabaster, AL)**. All cell lines were grown at 37°C under humidified air containing 5% CO_2_. A549, PC-3, DU145 and H1299 cells were maintained in RPMI-1640 medium supplemented with 2 mM L-glutamine, 1 mM sodium pyruvate and 10% (v/v) fetal bovine serum (FBS, Atlanta Biologicals, Atlanta, GA). HepG2 and HeLa cells were grown in Eagle's Minimum Essential Medium, while HCT116 and HCT116 *p53*^−/−^ cells were in McCoy's Medium, all supplemented with FBS to a final concentration of 10%.

### Transient transfection

Cells (2 x 10^6^) were transfected with specified expression vector (2 μg DNA) using a Neon transfection system (Invitrogen) according to the manufacturer’s protocol. Corresponding “empty” vectors were used as controls. pEGFP-N1/CerS6 vector for expression of GFP-CerS6 fusion was constructed by subcloning the CerS6 coding sequence from the pCMV/CerS6 vector used in our previous study [26] into pEGFP vector (Addgene, Cambridge, MA) downstream of GFP. pCMV6-AC-RFP/CALR and pCMV6-AN-RFP/MAP1LC3B vectors were purchased from OriGene (Rockville, MD).

### siRNA

The siRNA knockdown of CerS6 or CerS4 was performed using siRNA duplexes purchased from Qiagen (Venlo, The Netherlands) (Table 1). Cells (∼1.5 × 10^5^) were transfected with 25 pmol of CerS6 or CerS4 siRNA using 5–10 μl of Lipofectamine 2000 (Invitrogen), according to manufacturer’s protocol. Scrambled siRNA with medium GC content (Invitrogen) was used as control.

**Table 1:** Primers used for siRNA silencing.

CerS4 Sense	r(GGA CAU UCG UAG UGA UGU A)dTdT
CerS4 Antisense	r(UAC AUC ACU ACG AAU GUC C)dTdT
CerS6 Sense	r(CGC UGG UCC UUU GUC UUC A)dTdT
CerS6 Antisense	r(UGA AGA CAA AGG ACC AGC G)dTdT

### Cell proliferation assays

Cell viability was assessed using an MTT (3-[4,5-dimethylthiazol-2-yl]-2,5-diphenyl tetrasodium bromide) cell proliferation assay (Promega, Madison, WI). Cells were plated at a density of 5 × 10^3^ cells/well in 96-well format, and MTT was added at specified time points. Plates were further processed according to the manufacturer's instructions. *A*_570 nm_ was measured using a Wallace 1420 multilabel counter (PerkinElmer Life Sciences, Waltham, MA).

### Western blot analysis

Cell lysates for western blot analysis were prepared in a buffer containing 50 mM Tris-HCl (pH 8.0), 150 mM NaCl, 2 mM EDTA, 1% Triton X-100, 0.1% SDS, 1 mM dithiothreitol, 1 mM PMSF and protease inhibitor cocktail (Sigma). Sample lysates were subjected to SDS-PAGE followed by immunoblot with corresponding antibodies. CerS6 and CerS4 polyclonal antibodies (1:1000) were purchased from Novus Biologicals (Littleton, CO). p53 was detected using in-house p53 monoclonal Ab (1:500). Actin was detected using a monoclonal antibody from Sigma (clone AC-15, 1:5000).

### Confocal microscopy

For live-cell confocal imaging, cells co-transfected with pEGFP/CerS6 and pCMV6-AC-RFP/CALR or pCMV6-AN-RFP/MAP1LC3B vectors were grown in 35-mm glass bottom microwell petri dishes (MatTek corporation, Ashland, MA). Images were captured using confocal laser scanning microscope Olympus FluoView FV10i (Olympus, Tokyo, Japan). Pearson’s coefficient for co-localization was calculated using Fiji software (figi.sc).

### LC-MS/MS analysis of sphingolipids

Approximately 1.5 × 10^6^ cells were trypsinized and washed twice with cold PBS. Samples were centrifuged at 1000 rpm for 5 min at 4°C, and the final pellet was stored at −80°C prior to analysis. Further preparation of samples and measurement of endogenous ceramides by LC-MS/MS followed the protocol described previously [29]. Briefly, samples were fortified with internal standards, and 2 ml of isopropyl alcohol: water: ethyl acetate (30:10:60; v:v:v) was added to the cell pellet mixtures. Samples were subjected to two rounds of vortex and sonication followed by a 10-min centrifugation at 4000 rpm. The supernatant or top layer was used as lipid extract and subjected to LC-MS/MS for analysis of ceramide species. Samples were normalized to total phospholipid inorganic phosphate (P_i_) present in the extract. Lipid extraction and analyses were performed by the Medical University of South Carolina Lipidomics Core facility (Charleston, SC).

### Statistical analysis

For statistical analysis of differences between two groups Student’s *t* test was performed using a GraphPad software. For the statistical analysis of differences between three groups one-way ANOVA was used.

## Results

### Antifolate MTX leads to the elevation of CerS6 and ceramides in cancer cell lines

**Fig 1:**
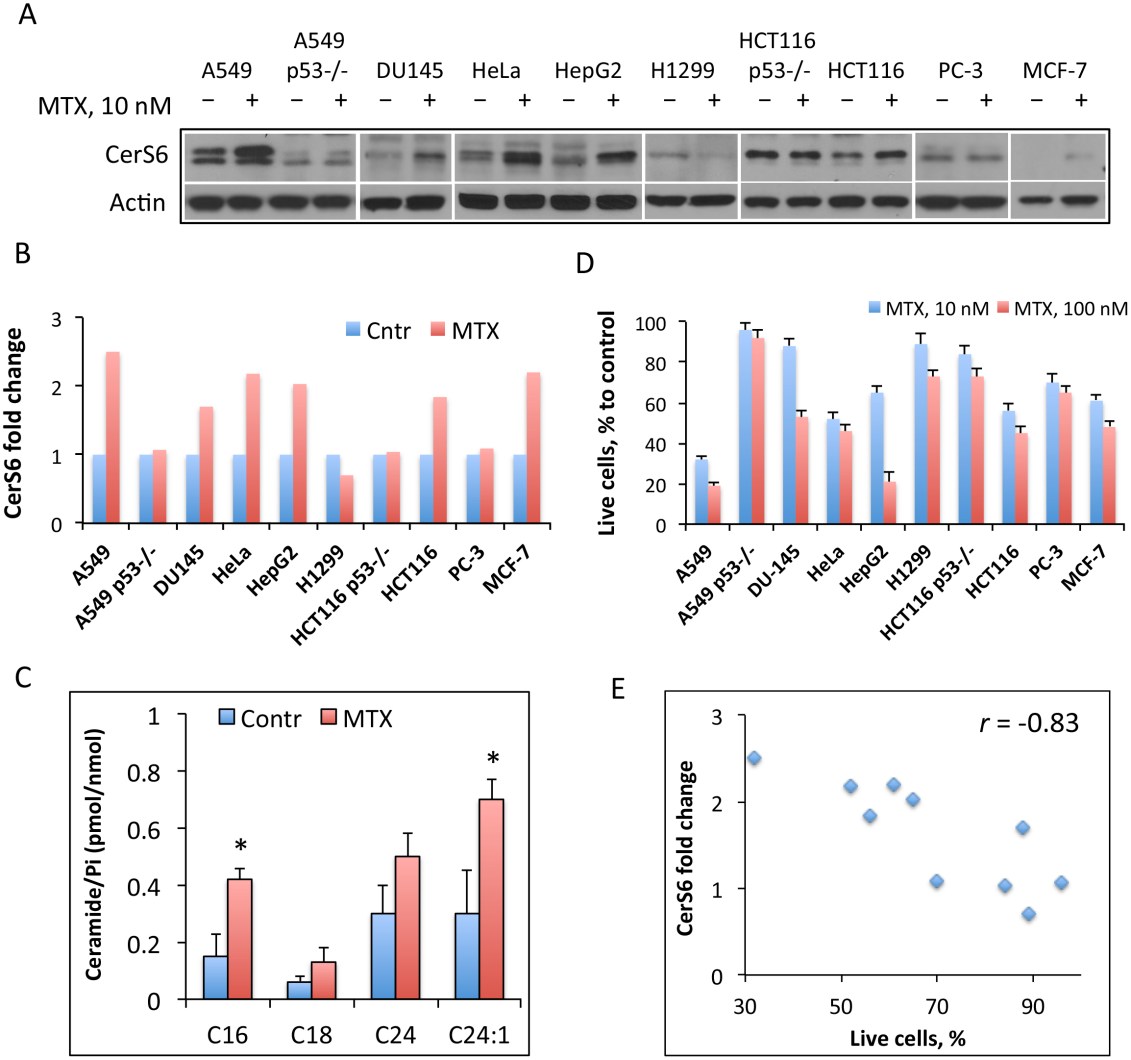
Effects of MTX on CerS6, ceramide levels and cellular proliferation. (A) Levels of CerS6 (Western blot) in cancer cells with and without MTX treatment. Cells were collected 48 h after MTX was added. (B) Changes in CerS6 levels after treatment with 10 nM MTX for 48 h (calculated from A). The intensity of bands was quantified using ImageJ software. In all cases, normalization for levels of actin was performed. (C) Changes in levels of selected ceramides in A549 cells after treatment with 10 nM MTX; P_i,_ total phospholipid inorganic phosphate present in cell extracts. Student’s *t*-test was performed for statistical analysis of the changes in ceramide levels and asterisks indicate statistically significant differences (p<0.05). (D) Antiproliferative effect of MTX (10 and 100 nM) in a panel of cancer cell lines. Live cells were assessed by MTT assay. Error bars show SD of four measurements. Differences between control and drug treated groups were statistically significant (calculated by one-way ANOVA) with *p* value below 0.0007 in all cases with the exception of A549 p53^-/-^ cells (p = 0.0357). (E) Scatter plot of CerS6 protein levels (from panel B) *versus* cell survival after MTX treatment (from panel D) for ten cell lines indicated in the above panels. Pearson’s coefficient (*r* = −0.83; n = 10; *p*<0.01) indicates strong negative correlation between changes in CerS6 levels and cell survival upon MTX treatment.

We have previously demonstrated that folate stress induced by the transient expression of the folate stress enzyme ALDH1L1 produces antiproliferative effect in two cell lines, A549 and HCT116 [26], by activating the expression of CerS6. This effect was also observed in the former cells upon folate withdrawal from culture medium [26]. Here we investigated whether common antifolate MTX produces the same effect. For this study, cell lines with different p53 status have been selected because previously we demonstrated the p53-dependent effect of folate stress on CerS6 [26]. We have observed that after treatment of a panel of 10 cancer cell lines with 10 nM MTX (near IC50 for DHFR) the elevation of CerS6 protein took place in six cell lines (Fig 1A and 1B). Of note, levels of CerS6 vary significantly between different cell lines (Fig 1A). In agreement with the elevation of CerS6, the increased levels of C_16_-ceramide, the product of CerS6 catalysis, were measured after MTX treatment of A549 cells (Fig 1C). We also observed that other ceramide species were elevated in cells exposed to the drug (Fig 1C) but the elevation of C_16_-ceramide (2.8-fold) was stronger than in case of other ceramides (2.3-, 1.7- and 2.3-fold for C_18_-, C_24_- and C_24:1_-ceramide, respectively). The evaluation of the antiproliferative effect of MTX in tested cell lines indicated that there was a correlation between CerS6 elevation and the potency of the drug: cell lines with more robust elevation of CerS6 were also more sensitive to MTX (Fig 1D). Pearson’s correlation was run to determine the relationship between changes in CerS6 levels and cell survival after MTX treatment, and a strong negative correlation was confirmed for these two parameters (r = −0.8297, Fig 1E). It appears that increase in CerS6 levels upon MTX treatment is important for mediating of the drug effect rather than the absolute level of the protein. For example, HCT116 p53^-/-^ cells with relatively high basal CerS6 level, which was not changed in response to MTX, were less sensitive to the drug than parental HCT116 p53^+/+^ cells with lower basal levels of CerS6, which were increased by about 1.9-fold in response to MTX (Fig 1B).

### CerS6 inhibits proliferation of MTX-sensitive cells and its loss protects against cytotoxic effect of the drug

**Fig 2:**
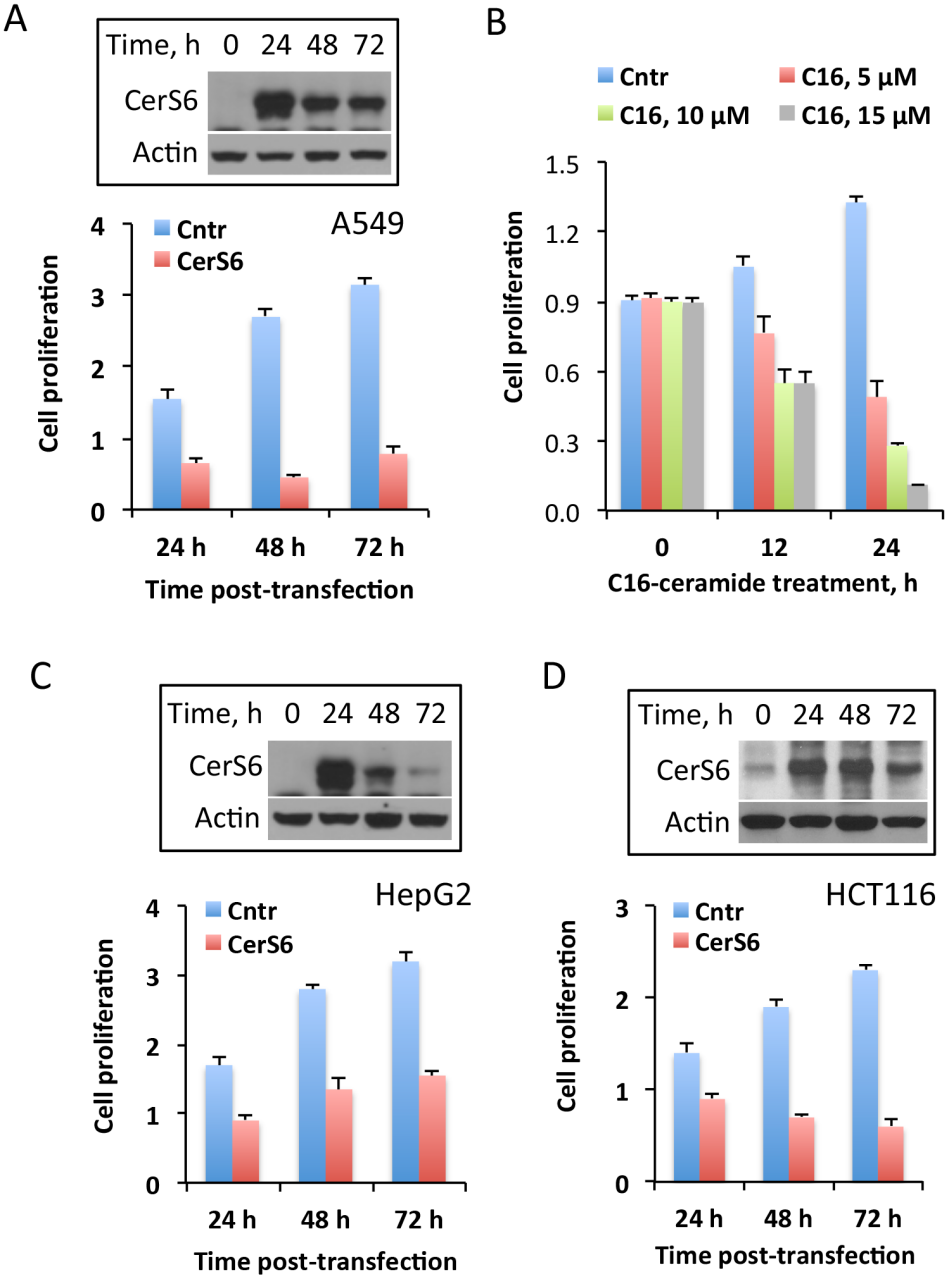
The effect of CerS6 transient expression on cancer cell lines highly sensitive to MTX. (A) Transient expression of CerS6 in A549 cells inhibits proliferation. (B) Antiproliferative effect of C_16_-ceramide on A549 cells. Live cells were assessed by MTT assay after exposure to C_16_-ceramide for indicated time; stock solution of ceramide (10 mM) was prepared in 50:50 mixture of DMSO/methanol and was added directly to cultured cells; control cells were treated with the same volume of DMSO/methanol mixture. Error bars show SD of four measurements. Differences between control and drug treated groups were statistically significant (calculated by one-way ANOVA) with *p* value below 0.0009. (C and D) Transient expression of CerS6 in HepG2 or HCT116 cells inhibits proliferation. For panels A, C, and D cells were transiently transfected with the CerS6 expression vector and proliferation was assessed by MTT assay at indicated time points. Data from MTT assay are plotted as fold change of live cells compared to control (number of cells at 0 h). Data show average of a representative experiment, six measurements for each data point, with error bars indicating SD; Student’s *t* test was performed for statistical analysis (in all data sets the difference between control and CerS6-transfected cells were statistically significant with *p<*0.005). Insets show levels of CerS6; actin was used as a loading control.

The elevation of CerS6 in response to MTX could be a non-specific off-target effect, which is not related to the drug cytotoxicity. Conversely, CerS6 itself could be a cellular stress inducer, with its activation by MTX being a part of this drug mechanism. Therefore, we asked the question of whether CerS6 elevation plays a role in the MTX antiproliferative effect. In support of the active role of CerS6 in MTX cytotoxicity, our experiments have shown that the elevation of CerS6 by transient transfection inhibited cellular proliferation in A549 cells (Fig 2A), indicating the role for the protein in antiproliferative cellular response. Most likely, this effect is associated with the increased production of C_16_-ceramide upon CerS6 elevation. In agreement with such mechanism, the treatment of A549 cells with C_16_-ceramide strongly inhibited their proliferation (Fig 2B). The antiptoliferative effect of CerS6 was further confirmed in two additional cell lines HepG2 (Fig 2C) and HCT116 (Fig 2D). Interestingly, it appears that the effect of CerS6 on cellular proliferation could be reversible. For example, HepG2 cells were able to partially restore their proliferation after CerS6 protein is no longer expressed (Fig 2C). The temporary inhibitory effect of CerS6 in this cell line was confirmed in an additional experiment, when we monitored these cells for 96 h CerS6 post-transfection: at this time point levels of CerS6 protein returned to normal and cells started to proliferate (data not shown). It is likely, though, that the extent of the CerS6 effect on proliferation could be cell-type specific so as the ability to clear the protein from the cell (Fig 2C, HepG2 cells demonstrated higher rate of CerS6 clearance compared to two other cell lines, Fig 2A and 2D).

**Fig 3:**
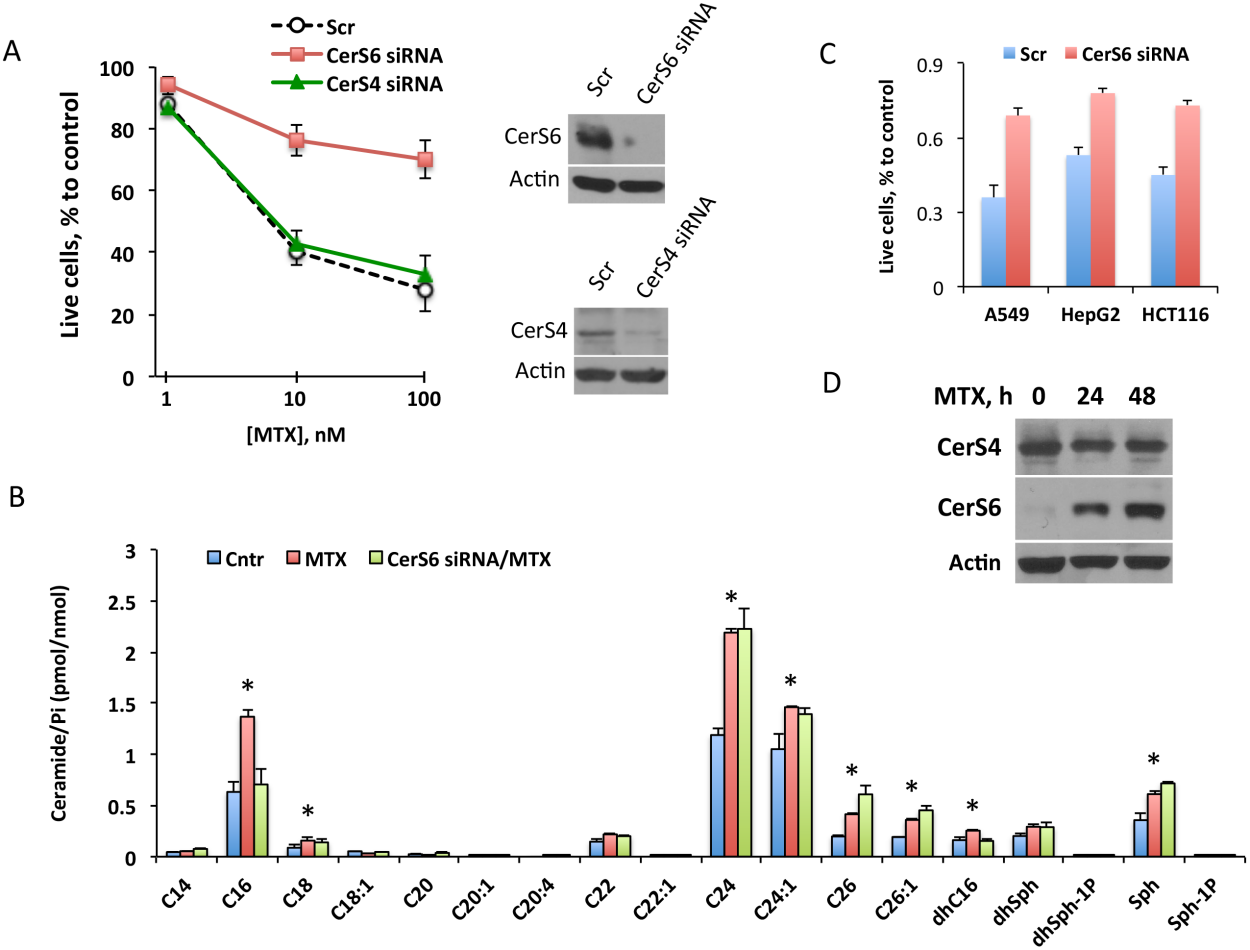
CerS6 is a target of MTX in mediation of antiproliferative effect. (A) Silencing CerS6 by siRNA rescues A549 cells from cytotoxic effect of MTX while silencing CerS4 does not change the drug toxicity. Cells were transfected with scrambled siRNA, CerS6 siRNA or CerS4 siRNA (25 pmol), MTX was added 6 h later and live cells were assessed by MTT assay 48 h after the beginning of MTX treatment. Data represent an average of two independent experiments, each performed in quadruplicates with error bars representing SD. Student’s *t* test was performed for statistical analysis (difference in cell number between control and CerS6-silenced cells were statistically significant with *p<*0.005; there were no significant differences in cell number between control and CerS4-silenced cells). Panels on the right show the efficiency of CerS6 and CerS4 silencing (evaluated at 48 h of MTX treatment). (B) Levels of ceramide in control A549 cells (*Cntr*, transfected with scrambled siRNA), MTX-treated cells transfected with scrambled siRNA (*MTX*) and MTX-treated cells after CerS6 silencing (*CerS6 siRNA/MTX*, transfected with CerS6 siRNA). Differences between the groups were analyzed by one-way ANOVA and asterisks indicate statistically significant changes (p<0.05). (C) Silencing of CerS6 by siRNA protects cancer cells from MTX cytotoxic effect. Student’s *t*-test was performed and the differences between MTX effect in control and CerS6-silenced cells were statistically significant (p<0.01). (D) MTX (10 nM) leads to elevation of CerS6 but not of CerS4 in A549 cells.

To study whether MTX cytotoxicity depends on CerS6, we have knocked down the protein in A549 cell using siRNA and evaluated the effect of the drug on CerS6-silenced cells. In these experiments, strong silencing of CerS6 has been achieved (Fig 3A). We observed that the loss of the enzyme significantly weakened the effect of MTX (Fig 3A) suggesting CerS6 as a mediator of the MTX response. In agreement with the role of CerS6 in C_16_-ceramide generation, this ceramide was not increased in response to MTX after CerS6 silencing (Fig 3B). At the same time, the effect of MTX on elevation of other ceramide species, notably C_18_-, C_24_- and C_24:1_-ceramide, was not attenuated by the CerS6 silencing (Fig 3B). The siRNA silencing of CerS6 in two other MTX-sensitive cell lines, HCT116 and HepG2, partially protected from MTX cytotoxic effect in a manner, similar to that in A549 cells (Fig 3C). In agreement with the effect of CerS6 through the elevation of C_16_-ceramide, treatment of CerS6-silenced cells with exogenous C_16_-ceramide strongly inhibited their proliferation (not shown), which is similar to the ceramide effect on non-silenced cells (Fig 2B).

**Fig 4:**
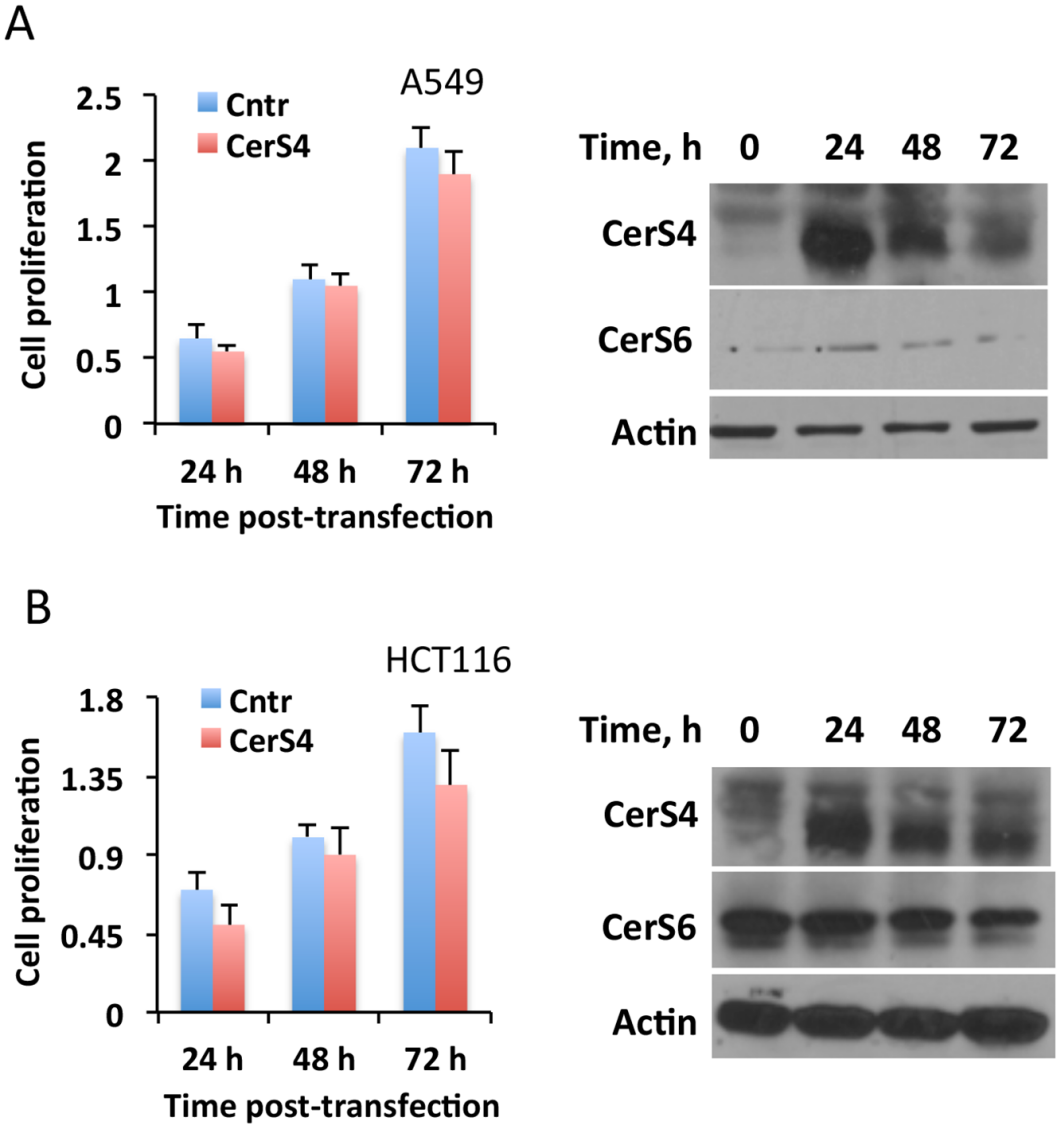
The effect of CerS 4 transient expression on cancer cell lines highly sensitive to MTX. Transient expression of CerS4 in A549 or HCT116 cells does not inhibit cellular proliferation (MTT assay). Data are average of two independent experiments, each done in quadruplicates with error bars representing SD. Differences between control and CerS4-expressing cells were not statistically significant (p>0.1) in both *panel A* and *panel B*. *Insets* show levels of CerS4 and CerS6 in the cells after transient expression of CerS4.

We have previously shown that another ceramide synthase, CerS4, could also be responsive to folate stress in terms of enhanced expression [26]. In this regard, the elevation of C_18_-ceramide, observed in cells treated with MTX (Fig 1D), would be in line with CerS4 elevation [30]. We observed that, unlike CerS6, CerS4 was not elevated upon MTX treatment of A549 cells highly sensitive to the drug (Fig 3D). To explore the potential role of CerS4 in the MTX effect directly, we have silenced it by siRNA (Fig 3A, Inset). The silencing of CerS4, however, did not protect cells from MTX cytotoxicity, a strict contrast to the CerS6 silencing (Fig 3A). Furthermore, while the elevation of CerS6 through a transient transfection induced strong antiproliferative effect in several cancer cell lines (Fig 2), the expression of CerS4 in our experiments had no noticeable effect on proliferation of A549 or HCT116 cells (Fig 4A and 4B). Of note, the transient expression of CerS4 did not have an effect on CerS6 level in A549 cells (Fig 4A) but resulted in its decrease in HCT116 cells (Fig 4B). Overall, these findings suggest that CerS6 is a target of MTX in the mechanism of action of this drug.

### Role of p53 in the effect of MTX on CerS6

**Fig 5:**
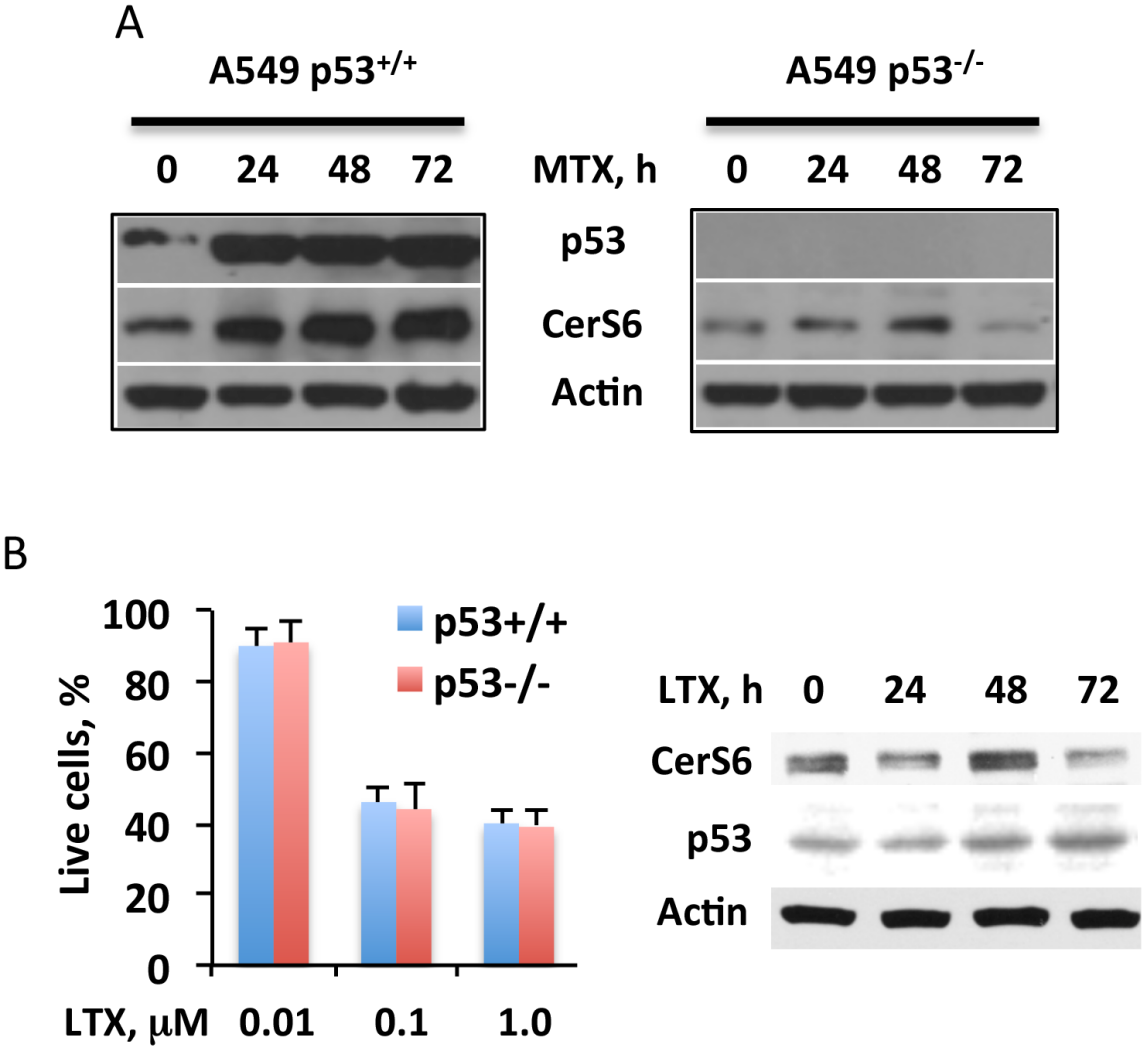
Targeting of CerS6 by MTX is p53-dependent. (A) MTX (10 nM) treatment strongly elevates CerS6 in p53^+/+^ but not p53^-/-^ cells. (B) Antifolate lometrexol (LTX) bypasses p53 and does not affect CerS6: *left panel*, LTX inhibits both p53^+/+^ and p53^-/-^ A549 cells (Experiments were performed three times with six wells per concentration point in each experiment; error bars, SD) no statistically significant differences between p53^+/+^ and p53^-/-^ cells were observed (p>0.1); *right panel*, levels of p53 and CerS6 (Western blot) in the cells after LTX treatment. Cells were exposed to LTX for 48 h.

We have previously shown that the CerS6 expression is activated upon transfection of cells with wild type p53 but not its mutant deficient in transcription regulation activity [26]. Overall, the study indicated that CerS6 is a likely transcriptional target for p53. Based on this, we have hypothesized that MTX activates CerS6 expression through a p53-dependent mechanism. Indeed, none of four tested p53^-/-^ cell lines (A549 p53^-/-^, H1299, HCT116 p53^-/-^ and PC-3) elevated CerS6 after MTX treatment while all five p53^+/+^ cell lines (A549, HeLa, HepG2, HCT116 and MCF-7) demonstrated such effect, though to a different magnitude (Fig 1A and 1B). In further support of the p53-dependent elevation of CerS6 by MTX, we have observed that the treatment of p53^+/+^ A549 cells with the drug strongly elevated p53 concomitantly with the elevation of CerS6 (Fig 5A, left panel). Of note, the MTX treatment resulted in only marginal (1.3-fold) CerS6 elevation in p53^-/-^ A549 cells at the 48 h time point (Fig 5A, right panel; the elevation of CerS6 in p53^+/+^ cells was about 3.4-fold). Interestingly, the treatment of A549 p53^+/+^ cells with another antifolate, lometrexol did not show a notable effect on CerS6 (Fig 5B; we did not consider a marginal elevation of CerS6 at 48 h time point as meaningful because its levels at 24 h and 72 h time points were slightly lower compared to the control; also, this apparent elevation was negligible, compared to that in p53-positive cells). It has been demonstrated, however, that the effects of this compound are p53-independent [31, 32]. In agreement with that early study, lometrexol did not result in the p53 elevation in A549 cells (Fig 5B). Furthermore, the silencing of p53 did not alter the cytotoxic effect of the drug (Fig 5B).

### Effect of MTX on subcellular localization of CerS6

**Fig 6:**
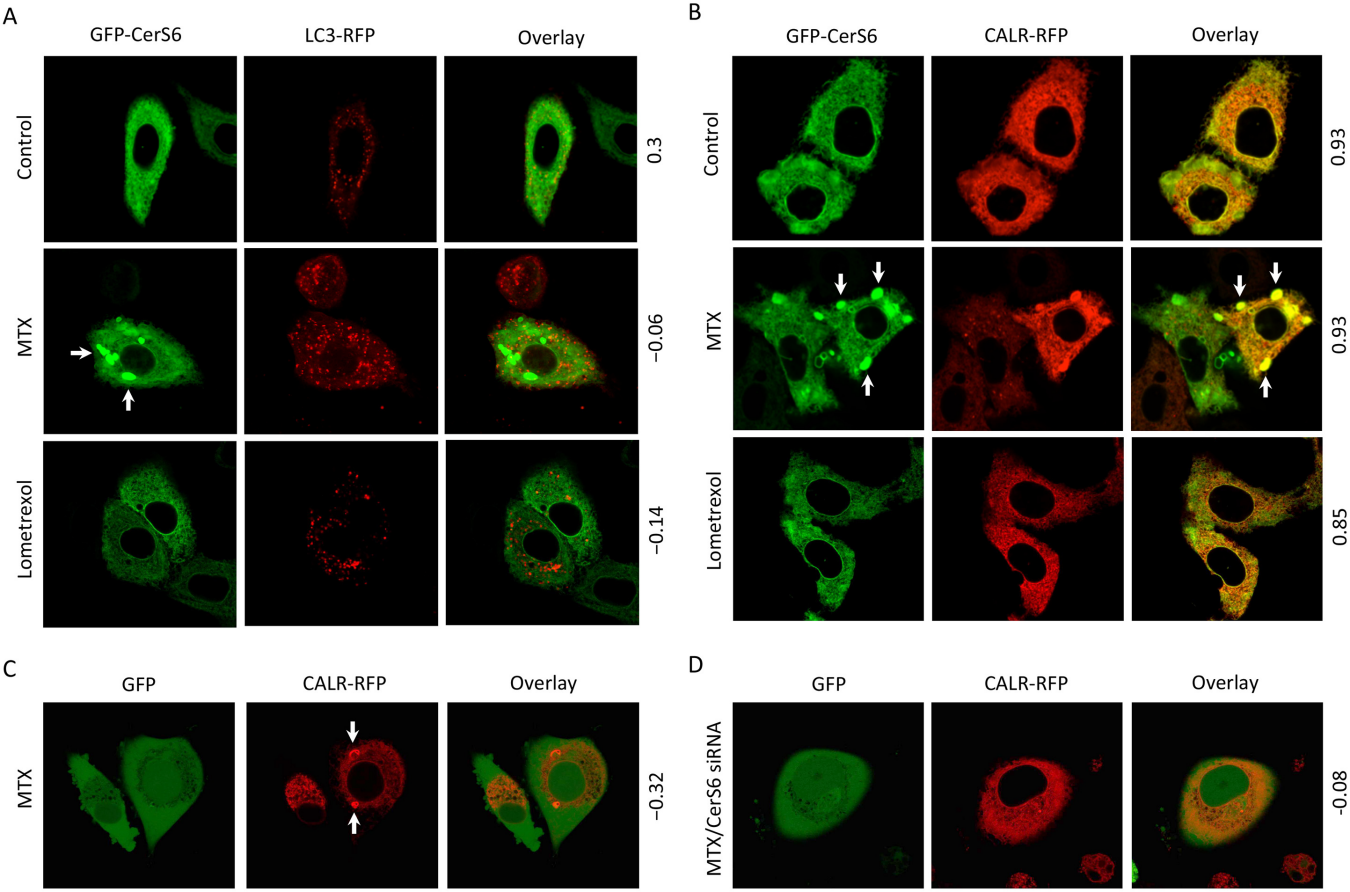
Methotrexate induces CerS6-dependent formation of ER membrane aggregates in A549 non-small cell lung carcinoma cells. (A) Treatment with MTX (10 nM) resulted in the increase of autophagosome number, but CerS6 was not associated with these organelles. (B) MTX (10 nM) induced aggregation of ER membranes and the accumulation of CerS6 in the aggregates. Arrows indicate ER membrane aggregates. Note the lack of such aggregates after lometrexol (1.0 μM) treatment. (C) MTX (10 nM) induced ER aggregation (detected by monitoring CALR-RFP) in the absence of exogenous CerS6. (D) A549 cells lacking CerS6 (siRNA silencing) did not form ER aggregates in response to MTX. Cells were co-transfected with GFP-CerS6 and LC3-RFP (panel A) or CALR-RFP (panel B), MTX was added 6 h after transfection and live cell images were captured 48 h later. In experiments with endogenous CerS6, cells were co-transfected with GFP and CALR-RFP. Six hours before co-transfection, cells were transfected with scrambled siRNA (*panel C*) or CerS6 siRNA (*panel D*). MTX was added 6 h after the second transfection and images were captured 48 h later. Pearson’s coefficient for co-localization (calculated using Fiji software) is indicated for each panel.

As the inhibitor of DHFR, with concomitant effects on folate metabolism, MTX might alter numerous downstream cellular processes. To study whether MTX can affect CerS6 subcellular localization, we have employed live-cell confocal imaging to monitor the GFP-CerS6 fusion transiently expressed in A549 cells. In these experiments, upon MTX treatment we have observed strong accumulation of CerS6 in vesicular structures reminiscent of autophagosomes while such vesicles were absent in control cells not treated with the drug (Fig 6A). Co-expression of GFP-CerS6 with RFP-fused LC3, the marker of autophagosomes [33], demonstrated the lack of their co-localization (Fig 6A) thus indicating that these vesicles were not autophagosomes. Of note, we observed the accumulation of autophagosomes in MTX-treated cells (Fig 6A). In contrast, co-expressed GFP-CerS6 and CALR-RFP (calreticulin, ER marker [34]) were co-localized in vesicles appeared upon MTX treatment (Fig 6B), indicating that these subcellular organelles are formed by ER membranes. The treatment of cells with lometrexol did not result in the formation of CerS6-containing ER aggregates even after treatment with high concentrations of the drug (up to 1.0 μM) (Fig 6B). The ER aggregates were observed only in MTX-treated cells, either naïve or transfected for GFP-CerS6 expression (Fig 6C and 6B) suggesting a specific role of the drug in this process. We next investigated whether CerS6 is required for the ER aggregation induced by MTX. To address this question, we exposed cells with siRNA-silenced CerS6 to the drug. In these experiments, CerS6 silencing almost completely prevented the aggregate formation (Fig 6D), the finding suggesting that MTX-induced ER aggregation is CerS6 dependent.

## Discussion

Our previous study demonstrated that ceramide pathways are responsive to the disruption of folate metabolism, induced by folate starvation or enzymatic alterations. Furthermore, we have identified CerS6 as a key ceramide synthase responding to intracellular folate alterations. Though mRNA levels of two ceramide synthases, CerS4 and CerS6, were noticeably increased in response to folate stress, CerS6 demonstrated much higher elevation than CerS4 [26]. Moreover, it appears that the prevention of CerS6 activation preserves cancer cells proliferation at conditions of folate stress. In line with these findings, the importance of CerS6 as a mediator of cell death in response to the treatment of BT474 cells with antifolate Pemetrexed in combination with sorafenib has been shown: the CerS6 siRNA silencing partially rescued drug treated cells [25]. In general, it is likely that CerS6 is a stress response protein, which translates various inputs to ceramide signaling. In support of this view, alterations in CerS6 levels or activity were reported in response to COX deficiency [35, 36], ER stress [37, 38], and ionizing radiation [39]. Furthermore, the protein is important for the mediation of cell death induced by such insults as photodynamic therapy [40], ER stress-inducing gene therapy [41], TRAIL [42] and chemotherapeutic drugs [25, 36], and also for the development-related apoptosis [43]. Such role of the protein is arguably associated with the production of C_16_-ceramide, which apoptotic effects have been well documented [44–47]. Curiously, though, the effect of CerS6 on gene expression, independent of its ceramide-generating function, has been reported [45]. Beyond the above effects, two recent studies pointed towards the role of CerS6 in energy expenditure, diet-induced steatohepatitis and insulin resistance thus underscoring it as a possible therapeutic target for treating metabolic diseases associated with obesity [48, 49]. These considerations prompted us to investigate whether CerS6 is a target for antifolate MTX, mediating the cellular response upon drug-induced disruption of folate metabolism.

Indeed, we have demonstrated that the MTX treatment of several cancer cell lines resulted in the elevation of CerS6 protein. Accordingly, the elevation of C_16_-ceramide, the main product of the enzyme [30, 50], was also observed in MTX-treated cells. In line with this observation, numerous studies indicate that C_16_-ceramide is the most relevant to alterations of CerS6 levels or activity [35–37, 46, 49, 51–53]. Of note, other ceramides were also elevated in response to MTX in a pattern reminiscent of that observed in studies of antifolate Pemetrexed [25]. These changes could be associated with the effect of CerS6 on other ceramide synthases, through a hetero dimerization or alterations of their expression [53, 54], but they also could be CerS6-independent and associated with additional MTX mechanisms. In support of this view, the loss of C_16-0_-dihydroceramide elevation, but not the elevation of ceramide species with a different acyl chain length, in response to Pemetrexed was observed after CerS6 silencing [25]. Interestingly, in our study stronger elevation of the enzyme was observed primarily in cell lines, which are more sensitive to MTX. The possibility that CerS6 is a mediator of MTX cytotoxicity, and not just an indicator of the change of protein signature, was suggested by the fact that CerS6 itself induces antiproliferative effects upon transient transfection. Our data also suggest that it is the elevation of CerS6 in MTX-sensitive cells that is important for the stress response rather than endogenous protein levels. Remarkably, the siRNA silencing of CerS6 strongly protected several cell lines from the MTX cytotoxic effect. Of note, another ceramide synthase, CerS4, which was elevated at the mRNA levels in response to ALDH1L1 or folate withdrawal, was not involved in the mediation of the MTX toxicity. Curiously, CerS4 was also not cytotoxic to cells after its elevation by transient transfection.

Our study further indicated that the elevation of CerS6 in response to MTX was p53 dependent. While the role of p53 in mediation of the MTX cytotoxic effect is not completely clear, several studies indicated the p53-dependent effect of the drug [12, 55, 56]. Accordingly, we have observed the elevation of p53 together with CerS6 in response to MTX. Our previous study indicated that the *CerS6* gene is likely a transcriptional target of p53 [26] and we suggest that MTX elevates CerS6 by activating p53. In agreement with the p53-dependent transcriptional mechanism of CerS6 elevation, its levels were barely changed in p53-deficient cells exposed to MTX. In line with these findings, an antifolate with p53-independent function, lometrexol [31, 32], failed to elevate CerS6. Curiously, in response to MTX CerS6 was also elevated in DU145 cells possessing two different allele-specific p53 mutants [57, 58]. Each of these mutants might retain partial transcription regulation activity [57, 58] but it is not clear at present whether CerS6 elevation in DU145 cells was associated with the function of mutant p53 or not.

**Fig 7:**
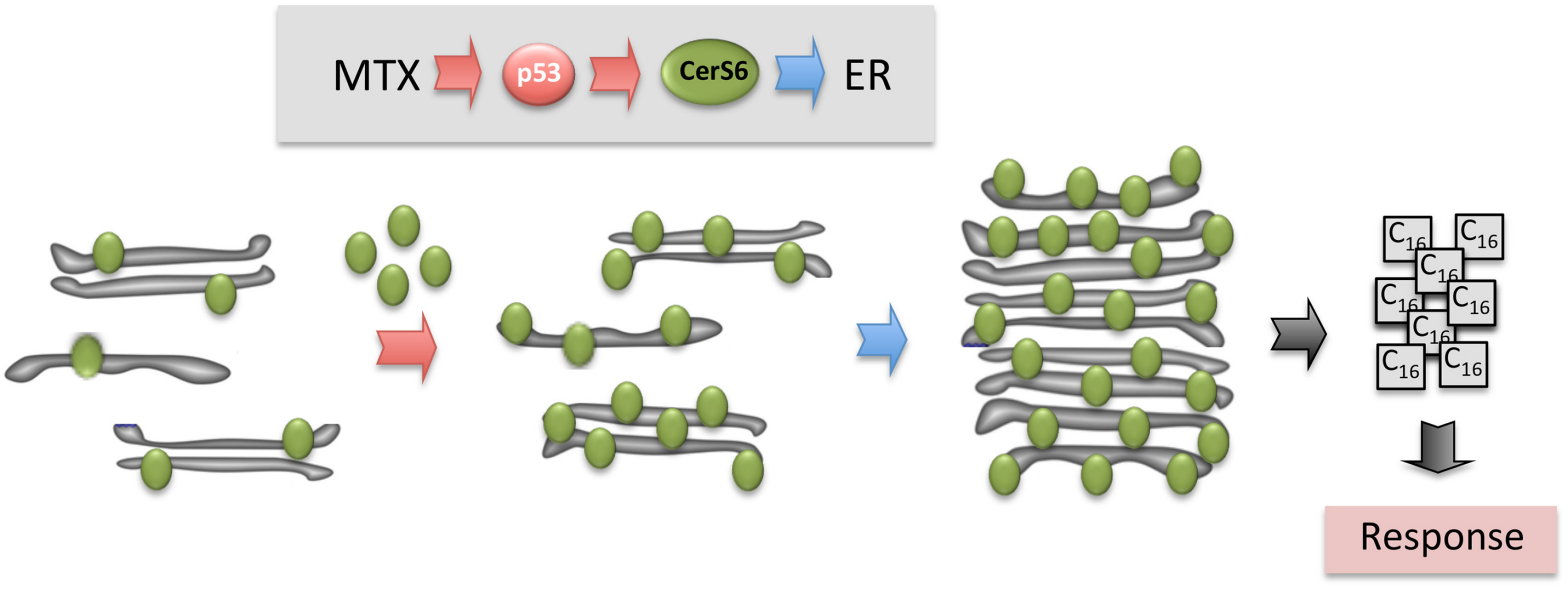
Proposed two-step mechanism for CerS6-dependent MTX cytotoxicity. Step 1: MTX induces p53, which functions as the transcriptional activator of CerS6 and increases levels of the protein in ER. Step 2: MTX induces ER stress leading to aggregation of CerS6-containing ER membranes. Enhanced generation of C_16_-ceramide by CerS6 further promotes cytotoxic effect.

MTX treatment also resulted in the accumulation of CerS6 in subcellular organelles reminiscent of autophagosomes. While CerS6 is an endoplasmic reticulum (ER) resident protein [50], its role in autophagosome formation has been reported [59]. In fact, autophagy is a common mechanism associated with ceramide-dependent cytotoxicity [23, 60]. For example, induction of autophagy appears to be a common response to pyridinium ceramides [61–63]. Interestingly, the effect of CerS6 on autophagosomes was seen in response to treatment with the combination of two drugs, one of which was antifolate Pemetrexed [59]. While MTX is not typically viewed as an inducer of autophagy, we have observed increased number of autophagosomes in cells treated with the drug in our study. The observed organelles with accumulated CerS6, however, were not autophagosomes but aggregates of ER membrane as was judged by their co-localization with the ER marker calreticulin and the absence of co-localization with the autophagosome marker LC3. Such aggregation has been suggested as an ER stress response different from the unfolded protein response and was seen upon treatment with different classes of pharmaceuticals [64, 65]. Of note, increasing ER size through membrane synthesis is a part of the cellular program to overcome ER stress [66]. We suggest that this process, leading to the formation of CerS6-containing ER aggregates, took place in MTX-treated cells in our experiments. Importantly, in our study the ER aggregation induced by MTX was CerS6 dependent, with CerS6 silencing significantly decreasing the aggregate formation. While MTX activates various down-stream pathways, it has been reported that sustained ER stress is the predominant mechanism behind the synergistic induction of cell death by this drug in the combination with AMPK activator, AICAR [67]. Interestingly, it has been suggested that the ER stress-regulated switch from pro-apototic response to protective autophagy could be involved in the MTX resistance in some cases of choriocarcinoma [68]. Based on our findings, we propose that the MTX treatment elevates CerS6 in a p53-dependent manner; this elevation produces ER-stress reflected by the accumulation of CerS6-containing ER aggregates; and p53-CerS6-ER associated mechanism is a component of the MTX-induced cytotoxicity (Fig 7).

## Conclusions

In summary, our study demonstrated that CerS6 is a target of antifolate MTX, predominantly in p53-positive cancer cell lines. In general, numerous classic chemotherapeutic drugs induce cell death by targeting ceramide metabolism [69] but such mechanism was not know for MTX. An intriguing finding that MTX induces re-organization of the ER membrane underscores a novel mechanism for the action of this drug. The finding that CerS6 is strongly localized to these aggregates, while the protein silencing decreased the aggregate formation, indicates a role of the protein in ER-dependent drug response (schematically depicted in Fig 7).
